# A study of generative large language model for medical research and healthcare

**DOI:** 10.1038/s41746-023-00958-w

**Published:** 2023-11-16

**Authors:** Cheng Peng, Xi Yang, Aokun Chen, Kaleb E. Smith, Nima PourNejatian, Anthony B. Costa, Cheryl Martin, Mona G. Flores, Ying Zhang, Tanja Magoc, Gloria Lipori, Duane A. Mitchell, Naykky S. Ospina, Mustafa M. Ahmed, William R. Hogan, Elizabeth A. Shenkman, Yi Guo, Jiang Bian, Yonghui Wu

**Affiliations:** 1 Department of Health Outcomes and Biomedical Informatics, College of Medicine, University of Florida, Gainesville, FL USA; 2 Cancer Informatics Shared Resource, University of Florida Health Cancer Center, Gainesville, FL USA; 3 NVIDIA, Santa Clara, CA USA; 4 Research Computing, University of Florida, Gainesville, FL USA; 5 Integrated Data Repository Research Services, University of Florida, Gainesville, FL USA; 6 Lillian S. Wells Department of Neurosurgery, Clinical and Translational Science Institute, University of Florida, Gainesville, FL USA; 7 Division of Endocrinology, Department of Medicine, College of Medicine, University of Florida, Gainesville, FL USA; 8 Division of Cardiovascular Medicine, Department of Medicine, College of Medicine, University of Florida, Gainesville, FL USA

**Keywords:** Translational research, Health care

## Abstract

There are enormous enthusiasm and concerns in applying large language models (LLMs) to healthcare. Yet current assumptions are based on general-purpose LLMs such as ChatGPT, which are not developed for medical use. This study develops a generative clinical LLM, GatorTronGPT, using 277 billion words of text including (1) 82 billion words of clinical text from 126 clinical departments and approximately 2 million patients at the University of Florida Health and (2) 195 billion words of diverse general English text. We train GatorTronGPT using a GPT-3 architecture with up to 20 billion parameters and evaluate its utility for biomedical natural language processing (NLP) and healthcare text generation. GatorTronGPT improves biomedical natural language processing. We apply GatorTronGPT to generate 20 billion words of synthetic text. Synthetic NLP models trained using synthetic text generated by GatorTronGPT outperform models trained using real-world clinical text. Physicians’ Turing test using 1 (worst) to 9 (best) scale shows that there are no significant differences in linguistic readability (*p* = 0.22; 6.57 of GatorTronGPT compared with 6.93 of human) and clinical relevance (*p* = 0.91; 7.0 of GatorTronGPT compared with 6.97 of human) and that physicians cannot differentiate them (*p* < 0.001). This study provides insights into the opportunities and challenges of LLMs for medical research and healthcare.

## Introduction

Generative large language models (LLMs) such as the ChatGPT^1^ have surprised the world by answering questions conversationally and generating textual content such as emails, articles, and even computer codes, triggering enormous enthusiasm in applying LLMs to healthcare^2–4^. People are enthusiastic about LLMs in the potential to facilitate documentation of patient reports (e.g., a progress report)^3,4^, improving diagnostic accuracy^5^, and assisting in various clinical care^6,7^, while at the same time concerning the hallucinations and fabrications^7,8^, bias and stereotype^9^, and risks of patient privacy and ethics^10^. Yet, this enthusiasm and concerns are based on ChatGPT, which is not designed for healthcare use^1^. Until now, it is unclear how this disruptive technology can help medical research and potentially improve the quality of healthcare.

Language model is a simple statistical distribution used in natural language processing (NLP) to formulate the probability of a sequence of words or the next word in a sequence. Surprisingly, when it is used as a learning objective to train a specific neural network architecture named transformer, and when the model size is very large such as billions or hundreds of billions of parameters, important artificial intelligence (AI) emerges. For example, LLMs can learn knowledge from one task and apply it to another task (i.e., transfer learning), learn from very few labeled samples (i.e., few-shot learning), and learn without human-labeled samples (i.e., zero-shot learning)^11–13^. The LLM pretrained using decoder-only transformer such as GPT-3 is known as generative LLM as it can generate human-like text. The conversational ability of LLMs is achieved using prompt-based text generation^14^, the key technology guiding LLMs to generate reasonable answers and contextual contents.

**Fig. 1:**
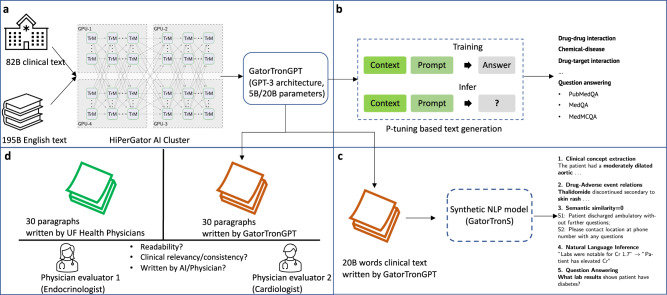
Develop a clinical generative large language model, GatorTronGPT, for biomedical natural language processing, clinical text generation, and healthcare text evaluation. **a** Train GatorTronGPT from scratch using GPT-3 architecture with up to 20 billion parameters. **b** Solve biomedical relation extraction and question answering using a unified P-tuning base text generation architecture. **c** Apply GatorTronGPT to generate 20 billion words of synthetic clinical text, which was used to train synthetic natural language processing model, GatorTronS. **d** Turing evaluation of 30 paragraphs of text written by GatorTronGPT mixed with 30 real-world paragraphs written by UF Health physicians. TrM transformer unit; B billion.

This study aims to develop a generative LLM using real-world clinical text and evaluate its utility for medical research and healthcare. We train GatorTronGPT using 82 billion words of de-identified clinical text^15^ from University of Florida (UF) Health and 195 billion diverse English words from the Pile^16^ dataset. We train GatorTronGPT from scratch using the GPT-3^17^ architecture. We formulate biomedical relation extraction and question answering using a unified text generation architecture^18^ to evaluate how GatorTronGPT could benefit medical research using 6 benchmark datasets. To examine the utility of text generation in the clinical domain, we apply GatorTronGPT to generate 20 billion words of synthetic clinical text, which are used to train synthetic NLP models using BERT^19^ architecture, denoted as GatorTronS (‘S’ stands for synthetic). We compare GatorTronS models with GatorTron^15^, a clinical NLP model trained using real-world 90 billion words of text, to test the hypothesis that generative clinical LLMs can be used to generate synthetic clinical text for medical research. To test if LLMs could be used in healthcare, two internal medicine subspecialists from endocrinology (NSO) and cardiology (MMA) manually evaluate clinical paragraphs written by GatorTronGPT compared with real-world paragraphs written by UF Health physicians. Figure 1 shows an overview of the study design. This study provides valuable insights into the opportunities and challenges of LLMs for medical research and healthcare.

## Results

### Training of GatorTronGPT from scratch

**Fig. 2:**
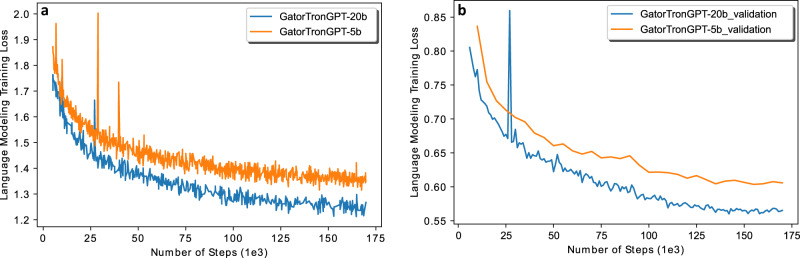
Training loss and validation loss for GatorTronGPT 5 billion and 20 billion models. **a** Training loss. **b** Validation loss.

Training the 5 billion GatorTronGPT model used approximately 6 days and the 20 billion model used about 20 days on 560 A100 80 G GPUs from 70 NVIDIA DGX nodes using the NVIDIA SuperPOD reference cluster architecture. Figure 2 shows the training and validation loss. Table 1 compares GatorTronGPT with GatorTronS and GatorTron on model architecture, training dataset, parameter size, and whether the model is a generative LLM, to help differentiate the three LLMs.

**Table 1:** Comparison of GatorTronGPT, GatorTronS, and GatorTron.

Model	Architecture	Training dataset	Parameters	Generative or not
GatorTronGPT	GPT3-based Decoder architecture	82 billion clinical words, 195 billion diverse English words	5 billion, 20 billion	Generative LLM
GatorTronS	BERT-based Encoder architecture	20 billion words of synthetic clinical text generated by GatorTronGPT	345 million	Non-generative LLM
GatorTron	BERT-based Encoder architecture	82 billion clinical words, 6 billion words from PubMed, 2.5 billion words from Wikipedia, 0.5 billion words from MIMIC III	345 million, 3.9 billion, 8.9 billion	Non-generative LLM

### GatorTronGPT for Biomedical natural language processing

Table 2a compares GatorTronGPT with four existing biomedical transformer models on end-to-end relation extraction of drug-drug interaction, chemical-disease relation, and drug-target interaction. GatorTronGPT outperformed all existing models, with the best F1-score of 0.500, 0.494, and 0.419, respectively. GatorTronGPT improved state-of-the-art by 3–10% compared with the second-best BioGPT^18^ model. We consistently observed performance improvement when scaling up the size of GatorTronGPT. Table 2b compares GatorTronGPT with six existing biomedical transformers using three benchmark datasets for biomedical question answering. The GatorTronGPT model with 20 billion parameters tied with BioLinkBERT on the MedQA dataset achieving the best performance of 0.451. GatorTronGPT also achieved the second-best performance of 0.776 for the PubMedQA dataset compared with the best performance of 0.782 from BioGPT. The performance of GatorTronGPT on the MedMCQA dataset was lower than a much larger LLM, Galactica, with 120 billion parameters.

**Table 2:** Comparison of GatorTronGPT with existing transformer models for (a) biomedical relation extraction and (b) question answering.

a									
	Biomedical Relation extraction								
	DDI			BC5CDR			KD-DTI		
Model	Pre	Rec	F1	Pre	Rec	F1	Pre	Rec	F1
GPT-2_medium	0.234	0.319	0.247	0.439	0.326	0.374	0.305	0.279	0.285
REBEL	0.354	0.286	0.283	0.343	0.395	0.367	0.324	0.296	0.304
REBEL-pt	0.465	0.396	0.406	0.409	0.212	0.279	0.357	0.326	0.333
BioGPT	0.417	0.448	0.408	0.494	0.412	0.450	0.400	0.397	0.384
GatorTronGPT-5B	0.466	0.518	0.491	**0.587**	0.434	0.472	0.422	0.436	0.412
GatorTronGPT-20B	**0.476**	**0.521**	**0.500**	0.543	**0.499**	**0.494**	**0.422**	**0.440**	**0.419**

b			
	Question answering		
	PubMedQA	MedQA (USMLE)	MedMCQA
Model	Accuracy	Accuracy	Accuracy
PubMedBERT	0.558	0.381	NA
BioELECTRa	0.642	NA	NA
BioLinkBERT	0.702	**0.451**	NA
GPT-2	0.750	0.333	NA
BioGPT	**0.782**	NA	NA
Galactica_120B	0.776	0.444	**0.529**
GatorTronGPT-5B	0.758	0.402	0.358
GatorTronGPT-20B	0.776	**0.451**	0.429

The best evaluation scores are bolded.

*DDI* drug-drug interaction, *BC5CDR* BioCreative V chemical-disease relation, *KD-DTI* drug-target interaction, *B* billion parameters, *NA* performance not reported.

### Evaluation of GatorTronS

**Fig. 3:**
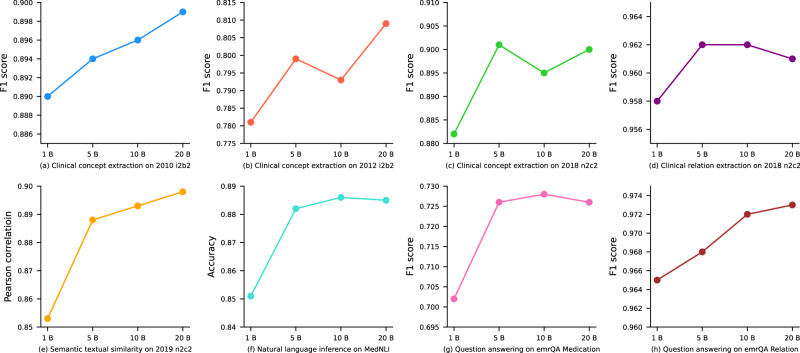
Comparison of GatorTronS models trained with 1, 5, 10, and 20 billion words of synthetic text on eight benchmark datasets. B billion words of text.

Tables 3 and 4 compare GatorTronS trained with different sizes of synthetic clinical text with ClinicalBERT and GatorTron^15^. For clinical concept extraction, GatorTronS, trained using 20 billion and 5 billion synthetic clinical text, achieved the best F1-score for the three benchmark datasets. GatorTronS outperformed the original GatorTron model by >1% F1-score on all three benchmark datasets. For medical relation extraction, the GatorTronS trained using 10 billion synthetic clinical text achieved the best F1-score of 0.962 on the 2018 n2c2 challenge benchmark dataset, which is comparable with the original GatorTron model (0.960). For semantic textual similarity and natural language inference, GatorTronS achieved the best evaluation scores, outperforming the original GatorTron by >1%. For question answering using emrQA dataset, GatorTronS outperformed the original GatorTron model trained using real-world clinical text by >1%. The comparison results show that a minimum of 5 billion words of synthetic clinical text are required to train a synthetic model with comparable performance to GatorTron, a transformer trained using 82 billion words of real-world UF Health clinical text. Figure 3 compares GatorTronS models trained with different sizes of synthetic text using line plots. We observed consistent performance improvements from all eight datasets by increasing the size of synthetic text from 1 billion to 5 billion words. The improvements are not consistent when increasing the data size from 5 billion up to 20 billion words.

**Table 3:** Comparison of GatorTronS with existing transformer-based LLMs for clinical concept extraction and medical relation extraction.

	Clinical concept extraction									Medical relation extraction		
	2010 i2b2^20^			2012 i2b2^21^			2018 n2c2^22^			2018 n2c2^22^		
Transformer	Precision	Recall	F1 score	Precision	Recall	F1 score	Precision	Recall	F1 score	Precision	Recall	F1 score
ClinicalBERT	NA	NA	0.878	NA	NA	0.789	0.859	0.883	0.871	0.968	0.941	0.954
GatorTron, 90B	0.875	0.904	0.889	0.764	0.822	0.792	0.876	0.904	0.890	0.972	0.948	0.960
GatorTronS, 1B	0.874	0.907	0.890	0.753	0.812	0.781	0.871	0.892	0.882	0.971	0.945	0.958
GatorTronS, 5B	0.879	0.909	0.894	0.777	0.823	0.799	**0.899**	0.903	**0.901**	0.974	0.949	**0.962**
GatorTronS, 10B	0.882	**0**.**911**	0.896	0.765	0.823	0.793	0.887	0.904	0.895	0.974	**0.950**	**0.962**
GatorTronS, 20B	**0.889**	**0.911**	**0.899**	**0.784**	**0.836**	**0.809**	0.892	**0.907**	0.900	**0.975**	0.947	0.961

B billion words of text Clinical concepts in 2010 i2b2 and 2012 i2b2 challenges: problems, treatments, lab tests; clinical concepts in 2018 n2c2 challenge: drugs, adverse events, and drug-related attributes (e.g., dose). Medical relation in 2018 n2c2 challenge: drug induced adverse events; B: billion words of text. Best evaluation scores are bolded. NA: scores not reported.

**Table 4:** Comparison of GatorTronS with existing transformer-based LLMs for semantic textual similarity, natural language inference, and question answering.

	Semantic textual similarity	Natural language inference	Question answering			
	2019 n2c2^23^	MedNLI^24^	emrQA Medication^25^		emrQA Relation^25^	
Transformer	Pearson correlation	Accuracy	F1 score	Exact Match	F1 score	Exact Match
ClinicalBERT	0.879	0.827	0.691	0.241	0.931	0.853
GatorTron, 90B	0.881	0.867	0.718	0.298	0.954	0.903
GatorTronS, 1B	0.853	0.851	0.702	0.288	0.965	0.924
GatorTronS, 5B	0.888	0.882	0.726	0.305	0.968	0.926
GatorTronS, 10B	0.893	**0.886**	**0.728**	**0.311**	0.972	**0.929**
GatorTronS, 20B	0.898	0.885	0.726	0.307	**0.973**	0.927

B: billion words of text. The best evaluation scores are bolded.

### Physicians’ Turing test

The Turing test results show that, on average, less than half (49.2%) of the clinical notes were identified correctly, including 36.7% of the synthetic notes and 61.7% of the human notes (Table 5a). Among the 30 synthetic notes written by GatorTronGPT, 9 (30.0%) and 13 (43.4%) were correctly labeled as ‘AI’ by the two physicians, respectively. Among the 30 human notes written by physicians, 17 (56.7%) and 20 (66.7%) were correctly labeled as ‘Human’, respectively. Considering GatorTronGPT was considered as a human for more than 30% of the instances (the criteria from Turing test)^20^, GatorTronGPT passed the Turing test (*p* < 0.001). Table 5b summarizes the means and standard deviations of the linguistic readability and clinical relevance and consistency. Statistical tests show that there is no significant difference between notes written by GatorTronGPT and human physicians in both linguistic readability (*p* = 0.22) and clinical relevance and consistency (*p* = 0.91). Table 5c shows two examples written by GatorTronGPT; more examples are provided in Supplementary Table S1. Percent agreement and interrater reliability were found to be good or excellent, as summarized in Supplementary Tables S2 and S3.

**Table 5:** Turing test results.

a.			
	Ground truth		
	AI (*n* = 30)	Human (*n* = 30)	Total (*n* = 60)
Physician 1	9 (30.0%)	17 (56.7%)	26 (43.3%)
Physician 2	13 (43.3%)	20 (66.7%)	33 (55%)
Overall	11 (36.7%)	18.5 (61.7%)	29.5 (49.2%)
*p*-value	<0.001	=0.104	<0.001

b.			
	Ground truth		
	AI (n = 30)	Human (n = 30)	*p*-value
Readability	6.57 (1.22)	6.93 (1.09)	0.22
Clinical relevance	7.00 (1.23)	6.97 (1.07)	0.91

c.			
Example 1 (Passed Turing test): HISTORY OF PRESENT ILLNESS: Ms. [**NAME**] is a [**AGE**]-year-old female with prior history of left breast cancer who is referred to the [**LOCATION**] Clinic to discuss surgical management of her left lung nodule which was picked up on CT scanning for surveillance. The patient’s chest CT obtained at an outside institution showed an incidental finding of a left lower lobe pleural nodule, which she brought with her to clinic today for further review. Ms. [**Name**] reports to be doing well, with no change in her baseline level of shortness of breath, but denies any difficulty breathing, cough or hemoptysis. She feels essentially back to baseline and plays golf one day a week. Otherwise, Ms. [**NAME **] has no other pulmonary complaints and has never smoked tobacco or inhaled second hand smoke. She does report several years of intermittent reflux, which worsened after eating rough foods. She underwent an upper GI study for this, which showed a small hiatal hernia as well as a moderate-sized sliding hiatal hernia. She subsequently underwent an abdominal CT for evaluation for mesenteric ischemia and was felt to have a dilated pancreatic duct. Due to suspected head pathology on imaging (although the final report is pending), she underwent an MRI, which confirmed no pancreatic pathology. Given this, Ms. [**NAME **] has been trying to avoid fatty and fried foods, which has improved her reflux. Ms. [**NAME **] does report recent onset of sleep apnea, for which she is using CPAP every night for the last month. She tolerates this well without complaints of dry mouth, gasping for air, or undue sleepiness. Ms. [**NAME **] was seen in our outpatient [**LOCATION**] clinic on [**DATE**] at which time it was determined that in order to obtain a tissue diagnosis of this left lower lobe lesion a VATS approach would be utilized. She presents today for further pre-operative planning. Currently, Ms. [**NAME**] denies any pulmonary symptoms, fevers, chills, cough or weight loss. PAST MEDICAL HISTORY: 1. CARDIAC RISK FACTORS: Hypertension, Hyperlipidemia 2. CARDIAC HISTORY: None 3. OTHER PAST MEDICAL HISTORY: Left lower lobe pulmonary nodule			
Example 2 (Failed Turing test): Assessment: ICD-9-CM 1. Pain in joint, pelvic region and thigh, unspecified laterality 719.45 fentaNYL (DURAGESIC) 75 MCG/HR HYDROcodone-acetaminophen (VICODIN HP) 5-300 MG Tablet 2. Migraine NOS/not Intrcbl 346.90 SUMAtriptan Succinate 3. Bone Pain Arthritis 333.90 Dexamethasone Sodium Phosphate 4. Bipolar disorder 296.80 traZODone (DESYREL) 100 MG Tablet prazosin (MINIPRESS) 2 MG capsule carvedilol (COREG) 3.125 MG tablet isosorbide mononitrate (IMDUR) 30 MG CR tablet Refer to Psychiatry clopidogrel (PLAVIX) 75 MG tablet SUMAtriptan Succinate 5. ASTHMA UNSPECIFIED 493.90 albuterol (PROAIR HFA;VENTOLIN HFA) 108 (90 BASE) MCG/ACT inhaler 6. Major depressive disorder, single episode, unspecified 296.20 DULoxetine (CYMBALTA) 60 MG capsule Refer to Psychiatry amitriptyline (ELAVIL) 25 MG tablet traZODone (DESYREL) 100 MG Tablet 7. POST-SURGICAL VARICOSE VEINS of LOWER EXTREMITIES 454.9 fentaNYL (DURAGESIC) 75 MCG/HR 8. Other and unspecified hyperlipidemia 272.4 simvastatin (ZOCOR) 40 MG tablet COMPREHENSIVE METABOLIC PANEL 9. PND (post-nasal drip) 784.91 loratadine (CLARITIN) 10 MG tablet 10. Bipolar I disorder, single manic episode, unspecified 296.00 clonazePAM (KlonoPIN) 1 MG tablet Refer to Psychiatry 11. Allergic rhinitis 477.9 loratadine (CLARITIN) 10 MG tablet 12. Grief reaction 309.0 traZODone (DESYREL) 100 MG Tablet 13. Encounter for long-term (current) use of other medications V58.69 methocarbamol (ROBAXIN) 750 MG tablet COMPREHENSIVE METABOLIC PANEL 14. GERD (gastroesophageal reflux disease) 530.81 lansoprazole (PRE			

a. Number and percentage of correctly identified notes; *p*-values were calculated using Chi-squared test. b. Means and standard deviations of the quality measures; *p*-values were calculated using *T*-test. c. Two examples of synthetic clinical text generated by GatorTronGPT. The text generation stops at maximum 512 tokens. Pass Turing test: both physicians labeled as “Human”; Fail Turing Test: both physicians labeled as “AI”.

## Discussion

This study develops a generative clinical LLM, GatorTronGPT, using the GPT-3 architecture^13^ with 277 billion words of mixed clinical and English text. GatorTronGPT achieves state-of-the-art performance for four out of six biomedical NLP benchmark datasets. Our previous GatorTron^15^ model, trained using an encoder-only BERT architecture with 8.9 billion parameters, also achieved state-of-the-art performance on six clinical NLP benchmark datasets. The two studies demonstrate the benefit of LLMs for biomedical and clinical research. GatorTronGPT can generate synthetic clinical text for developing synthetic clinical NLP models (i.e., GatorTronS), which achieve better or comparable performance to GatorTron, an NLP model trained using real-world clinical text, demonstrating the utility of synthetic clinical text generation. The physicians’ Turing test show that GatorTronGPT can generate clinical text with comparable linguistic readability and clinical relevance to real-world clinical notes. This study provides valuable insights into the opportunities and challenges of generative LLMs for medical research and healthcare.

We discover an important utility of synthetic clinical text generation. To date, there has been a gap in accessing and sharing large-scale clinical text and clinical LLMs due to the sensitive nature of clinical text and the fact that automatic de-identification systems cannot remove 100% protected health information (PHI). Not surprisingly, a recent study^21^ on clinical foundation models point out that most LLMs in the medical domain are trained using “small, narrowly-scoped” clinical dataset with limited note types (e.g., MIMIC^22^) or “broad, public” biomedical literature (e.g., PubMed) that has limited insights to healthcare. Generative LLMs can provide large-scale synthetic clinical text to fill the gap. We compare the synthetic text with real-world clinical text to examine why GatorTronS, a transformer model trained using a much smaller (e.g., 5 billion words) synthetic clinical text corpus, could achieve better or comparable performance to GatorTron^15^, a transformer model trained using a much larger (90 billion words) real-world clinical text corpus. We identify potential reasons including (1) real-world clinical text has significant redundancies, which is a well-known characteristic of clinical narratives^23^, and (2) GatorTronGPT generates more diverse synthetic clinical text. We randomly sample a subset of real-world clinical notes with number of words comparable to the synthetic text (i.e., 20 billion words) to compare the coverage of unigrams (i.e., individual tokens) and bigrams (i.e., two consecutive tokens). The comparison results show that the synthetic text generated by GatorTronGPT contain remarkably more diverse unigrams (40.43 million : 4.82 million, ratios are reported as “synthetic” : “real notes”) and bigrams (416.35 million : 62.51 million); the synthetic text also has higher entropy than the real-world clinical text (4.97: 4.95). Supplementary Table S4 provides detailed comparison results and examples. A previous study^24^ has reported that by augmenting real-world clinical training data using additional human annotated synthetic text generated by a smaller generative LLM, GPT-2, NLP models can achieve better performance. Our study further demonstrates that, without additional human annotation and augmentation of training data, a larger clinical GPT-3 model can generate synthetic clinical text to train synthetic NLP models outperforming NLP models trained using real-world clinical text. Text generation using generative LLMs could mitigate the risk of exposing patient privacy and improve accessing and sharing of large-scale clinical text and NLP models, thus enabling the next generation of clinical text analytics using synthetic clinical text.

Generative LLMs aspire to become a “Unified Field Theory” to unify most fundamental NLP tasks using a single model architecture. It might be still early to judge if LLMs will become the one and only foundation model^12^ for NLP, but it looks like we are closer than ever. Generative LLMs have the potential to impact medical research in many aspects. In addition to performance improvement demonstrated in this study, generative LLMs provide a unified solution using prompt-based text generation^25^, which leads to a new paradigm of “one model for all NLP tasks” and has better few-shot learning and transfer learning ability to deliver portable clinical NLP systems^13,26^. The evaluation of GatorTronGPT shows that clinical LLMs can be used to generate clinical-relevant content with the potential to help document^3^ and code patient information in EHR systems, thus reducing the extensively onerous documentation burden for clinicians^27–29^. The prompt-based text generation of LLMs can potentially help compose treatment plans by integrating instructions from clinical guidelines and patients’ historical records in EHRs. The conversational ability of LLMs provides opportunities to develop intelligent EHR systems with human-like communication^2^, where healthcare providers, patients, and other stakeholders can communicate in an intelligent electronic health record (EHR) system. Industry stakeholders such as Epic and Nuance have been reported to be exploring these potentials^30,31^.

Our Turing test focuses on (1) linguistic readability; (2) clinical relevance; and (3) physicians’ ability to differentiate synthetic and human notes. The statistical tests show that there are no significant differences in linguistic readability (*p* = 0.22; 6.57 of GatorTronGPT compared with 6.93 of human) or clinical relevance (*p* = 0.91; 7.0 of GatorTronGPT compared with 6.97 of human). Further, physicians cannot differentiate them (*p* < 0.001), suggesting the potential utility of GatorTronGPT for text generation in healthcare. Two physician evaluators find that the texts written by GatorTronGPT generally lack clinical logic, indicating that more research and development are needed to make this technology mature for healthcare. Our Turing test focuses on statistical differences not utility in real-world clinical practice, which should be examined in future studies when this technology matures. A recent study^32^ examined an LLM developed at New York University, i.e., NYUTron, and our previously developed GatorTron^15^ for prediction of readmission, in-hospital mortality, comorbidity, length of stay, and insurance denial, demonstrating the potential utility of LLMs in healthcare.

While LLMs are promising for healthcare applications, much more research and development are needed to achieve this goal. Current general-purpose LLMs are designed for conversation as a chatbot outside of healthcare. Therefore, the current use of ChatGPT for healthcare is more like a typical case of intended use versus actual use as described in the medical device regulation^33^. Domain-specific LLMs are needed for clinical applications. Due to the noisy data and probabilistic nature of text generation, LLMs are prone to confabulation or hallucination, which is dangerous for healthcare. In this study, we adopted robust decoding strategies (e.g., nucleus sampling) to alleviate potential off-target text generation. Researchers are exploring solutions such as reinforcement learning from human feedback (RLHF)^34^ to reduce hallucinations, but it is still a not yet solved limitation of current LLMs. Future studies should explore strategies to better control the hallucinations at a minimal level to ensure the safety of using LLMs in healthcare. The security and risk of LLMs must be carefully examined in healthcare settings. We applied a de-identification system to remove PHIs from UF Health notes before training GatorTronGPT, future studies should carefully examine if GatorTronGPT has potential risk of speaking out PHIs and quantify the potential risk of re-identify real-world patients. Synthetic data, though generated by AI models, may still mirror the characteristics of its source material (e.g., UF health clinical notes). For example, ChatGPT has been reported to accidentally leak sensitive business data from a private company^35^. In addition, people are increasingly aware of the potential bias of AI applications in healthcare. Bias inherited from the original training data may be imitated and sometimes even amplified by AI models, which may cause systematic bias to specific patient groups^36^. Future studies should explore strategies to mitigate potential bias and ensure fairness of LLM applications. Like any medical AI applications, it is necessary to carefully examine this disruptive new technology to guide its application and make it “approved ” AI-enabled medical tool^37^.

## Methods

We developed GatorTronGPT using 82 billion words of de-identified clinical text^15^ from the University of Florida (UF) Health and 195 billion diverse English words from the Pile^16^ dataset. We trained GatorTronGPT from scratch using the GPT-3^17^ architecture (used by ChatGPT). We formulated biomedical relation extraction and question answering using a unified text generation architecture^18^ and evaluated GatorTronGPT using 6 biomedical benchmark datasets. To examine the utility of text generation, we applied GatorTronGPT to generate 20 billion words of synthetic clinical text, which were used to train synthetic NLP models, denoted as GatorTronS (“S” stands for synthetic). We compared GatorTronS with GatorTron^15^, a clinical NLP model trained with the same architecture but using real-world clinical text. To test if LLMs could generate text for healthcare settings, two internal medicine subspecialists from endocrinology (NSO) and cardiology (MMA) manually evaluated 60 clinical paragraphs including 30 paragraphs written by GatorTronGPT randomly mixed with 30 real-world paragraphs written by UF Health physicians. Figure 1 shows an overview of the study design.

### Data source

This study used 82 billion words of clinical narratives from UF Health Integrated Data Repository (IDR) and 195 billion words of diverse English words from the Pile^16^ corpus. This study was approved by the University of Florida Institutional Review Board under IRB202102223; the need for patient consent was waived. At UF Health, we collected approximately 290 million clinical notes from 2011–2021 from over 126 departments, approximately 2 million patients and 50 million encounters from inpatient, outpatient, and emergency settings^15^. We merged the UF Health clinical corpus with the Pile^16^ dataset to generate a large corpus with 277 billion words. We performed minimal preprocessing for the Pile dataset and applied a de-identification system to remove 18 PHI categories defined in the Health Insurance Portability and Accountability Act (HIPAA) from the UF Health notes.

### Preprocessing and de-identification of clinical text

Following our previous study^15^, we performed a minimal preprocessing procedure. First, we removed all empty notes and the notes with less than 10 characters followed by performing a deduplication at the note level using the exact string match strategy. Then, we leveraged an internally developed preprocessing tool (https://github.com/uf-hobi-informatics-lab/NLPreprocessing) to normalize the clinical text. The normalization processing consists of three steps including (1) unifying all text into UTF-8 encoding, removing illegal UTF-8 strings, and removing HTML/XML tags if any; (2) sentence boundary detection where we normalize the clinical notes into sentences; (3) word tokenization where we used heuristic rules to separate punctuation and special symbols (e.g., slash, parenthesis) from words (e.g., converting “(HbA1c)” to “(HbA1c)” and “excision/chemo” to “excision/chemo”) and fixing concatenations (e.g., missing white space like converting “CancerScreening ” to “Cancer Screening”). After preprocessing, we performed another deduplication at the sentence level using the exact string match strategy.

To de-identified the UF Health clinical notes, we adopted an internally developed de-identification system which consists of an LSTM-CRFs based model and a postprocessing module replacing system-detected protected health information (PHI) entities with dummy strings (e.g., replace patients’ names with [**NAME**]). We adopted the safe-harbor method to identify 18 PHI categories defined in the Health Insurance Portability and Accountability Act (HIPAA). The LSTM-CRFs model for PHI detection was trained using the publicly available 2014 i2b2 de-identification datasets and an internal dataset with over 1100 clinical notes from UF Health annotated for PHI removal (named as UF-deid-dataset; not publicly available due to IRB restrictions). After three years of continuous customization and improvement at UF Health, the current model achieved an overall F1 score of 97.98% (precision of 96.27% and recall of 99.76%) on the UF-deid-dataset test set, which means our de-identification system can remove 99.76% of all PHIs. Detailed information about the development of the de-identification system can be accessed from our previous paper^38^.

### Train GatorTronGPT from scratch

We trained GatorTronGPT using 5 billion parameters and 20 billion parameters and determined the number of layers, hidden sizes, and number of attention heads according to the guidelines for optimal depth-to-width parameter allocation proposed by ref. ^39^ as well as our previous experience in developing GatorTron^15^. The 5 billion model has 24 layers, hidden size of 4,096, and number of attention heads of 32; the 20 billion model has 44 layers, hidden size of 6144, and number of attention heads of 48. We trained the 5 billion model using a 2-way tensor model parallel with a batch size of 1120 and learning rate of 1.200E-05. We trained the 20 billion model using an 8-way tensor model parallel with a batch size of 560 and a learning rate of 1.000E-05. We adopted a dropout rate of 0.1. We inherited the GPT-3 architecture implemented in the MegaTron-LM^40^ and trained GatorTronGPT models from scratch with the default GPT-3 loss function^13^. We used a total number of 560 NVIDIA DGX A100 GPUs from 70 superPOD nodes at UF’s HiPerGator-AI cluster to train GatorTronGPT by leveraging both data-level and model-level parallelisms implemented by the Megatron-LM package^40^. (See https://github.com/NVIDIA/Megatron-LM for more details) We monitored the training progress by training loss and validation loss using 3% of the data and stopped the training when there was no improvement.

### GatorTronGPT for biomedical relation extraction and question answering

End-to-end relation extraction is an NLP task to identify the triplets <*concept1, concept2, relation*> from biomedical text. Question answering is to identify the *answer* for a given *question* and the *context*. Following previous studies^18,41^, we approached the two tasks using a unified prompt-based text generation architecture. Specifically, we adopted a fixed-LLM prompt-tuning strategy^42^ to attach a continuous embedding (i.e., virtue tokens) to the input sequence [*virtual tokens; x; y*] as a soft prompt to control the text generation; the LLM was not changed during training. We provide details in the Supplement.

#### End-to-end biomedical relation extraction

We compared the two GatorTronGPT models with four existing transformer models including GPT-2^43^, REBEL, REBEL-pt^25^, and BioGPT^18^ on three biomedical tasks for end-to-end relation extraction using three benchmark datasets including drug-drug interaction^44^ (DDI), BioCreative V chemical-disease relation^45^ (BC5CDR), and drug-target interaction^46^ (KD-DTI).

#### GPT-2

GPT-2 was trained using text data from 8 million webpages with 1.5 billion parameters, which is a scale-up of the first generation of GPT45 model. The GPT model outperformed previous transformer models on 9 out of 12 NLP tasks, whereas, the GPT-2 model further demonstrated text generation ability, which laid foundation for complex NLP tasks such as machine reading comprehension and question answering.

#### REBEL and REBEL-pt

REBEL is a transformer model based on the BART architecture designed for end-to-end relation extraction using sequence-to-sequence modeling, which outperformed previous relation extraction models based on classifications. REBEL-pt is an enhanced version of REBEL by further fine-tuning it using the triplets derived using Wikipedia hyperlinks.

#### BioGPT

BioGPT is a domain-specific generative transformer-based LLM developed using the GPT-2 architecture and the Pubmed biomedical literature, which achieved good performance in NLP tasks including relation extraction and question answering in the biomedical domain.

Following the previous study^18^, we formulated both biomedical relation extraction and question answering as a prompt-based text generation model and applied prompt-tuning (p-tuning) algorithms. We concatenate learnable soft prompts (also called virtual prompt embeddings) with the word embeddings from the *context* (i.e., input sentence). The sample sequence is constructed as [*prompt*, *context*, *relation*], where the prompt is generated using a LSTM model and the *relation* is the gold standard label including the head entity, tail entity, and their relation type. During the inference, the *context* and the *prompt* are used as the input for our GatorTronGPT model to condition and let the model generate the relations. We converted the original relation triplets into a sequence representation. For example, there is an “*agonist*” relation between a drug - “*Igmesine*” and a target “*Opioid receptor sigma 1*”, which was converted as: “the relation between [*Igmesine*] and [*Opioid receptor sigma 1*] is [*agonist*]*”*. Thus, the relation extraction can be solved as a text generation. During inference, we converted the generated text back to triplets for evaluation. We fine-tuned and evaluated our GatorTronGPT on the end-to-end relation extraction task across four biomedical datasets: BC5CDR (chemical–disease–relation extraction), KD-DTI (drug–target–interaction extraction), DDI (drug–drug–interaction extraction) and 2018 n2c2 (Drug-ADE-relation extraction). The precision, recall, and F1 score were used for evaluation.

#### Biomedical question answering

We compared GatorTronGPT with six existing transformer models using three widely used benchmark dataset including PubMedQA^47^—a biomedical question answering dataset collected from PubMed abstracts, which requires answering questions with ‘*yes/no/maybe*’ ; MedMCQA^48^—a large-scale multi-choice question answering dataset designed to address real world medical entrance exam questions covering 2400 healthcare topics and 21 medical subjects; and MedQA-USMLE^49^—a multi-choice dataset collected from the professional medical board exams. These datasets have been widely used to evaluate LLMs^18,47–49^.

Given a question, a context, and candidate answers, we concatenated the context and the candidate answers into a source sequence and compose the target sequence as: “the answer to the question given possible options is:”, “answer”: “C”. Then, we adopted soft prompts instead of hard prompts (manually designed clear text phrases) in p-tuning. Specifically, we used a randomly initiated continuous embedding as soft prompts, which were fine-tuned in the training. For the PubMedQA dataset, we explored the provided artificially generated text data. Specifically, we automatically labeled the generated text using our p-tuning model developed using the training set and experimented to feedback different proportion of auto-labeled data into training. The best performance was achieved by using 5% of the auto-labeled artificially generated text data. For p-tuning, we used the implementation in NVIDIA NeMo^50^, which is optimized for LLMs. We used the following parameters in our p-tuning: a global batch size of 32, virtual tokens for p-tuning 15, encoder MLP with encoder hidden size of 2048, max sequence length of 4096 for PubMedQA (long abstracts), 2048 for MedMCQA and MedQA-USMLE, and a fused Adam optimizer with a learning rate of 1e-4 and a weight decay of 0·01, betas of 0·9 and 0·98, a cosine annealing scheduler monitoring validation loss with a 50 step warm up. For example, the below is a prompt we used for MedQA-USMLE.

{“taskname”: “usmle-qa”, “prompt”: “QUESTION: A 23-year-old man comes to the physician for evaluation of decreased hearing, dizziness, and ringing in his right ear for the past 6 months. Physical examination shows multiple soft, yellow plaques and papules on his arms, chest, and back. There is sensorineural hearing loss and weakness of facial muscles bilaterally. His gait is unsteady. An MRI of the brain shows a 3-cm mass near the right internal auditory meatus and a 2-cm mass at the left cerebellopontine angle. The abnormal cells in these masses are most likely derived from which of the following embryological structures?\nMULTIPLE CHOICES: (A) Neural tube\n(B) Surface ectoderm\n(C) Neural crest\n(D) Notochord\nTARGET: the answer to the question given possible options is: “, “answer”: “C”}

#### GatorTronGPT for synthetic clinical text generation

We sought to test the hypothesis that LLMs can generate synthetic clinical text to train synthetic NLP models useful for medical research. We applied GatorTronGPT to generate synthetic clinical text according to a set of seeds without any fine-tuning, which is a typical zero-shot learning setting. Then, using the generated synthetic clinical text, we trained synthetic transformer-based NLP models using our previous BERT-based GatorTron architecture^15^, denoted as GatorTronS (‘S’ stands for synthetic). We trained GatorTronS models using different sizes of synthetic clinical text and compared them with the original GatorTron model trained using UF Health clinical text. To make it comparable, we trained GatorTronS using the same architecture and number of parameters (i.e., 345 million) as GatorTron^15^. We provide detailed information in the Supplement.

#### Synthetic clinical text generation

Following previous studies^51^, we approached synthetic clinical text generation using an iterative sampling algorithm and applied *top-p* (i.e., nucleus sampling) sampling and temperature sampling to balance the diversity and quality of text generation^51^. We approached the synthetic clinical text generation as an open-ended text-to-text generation task^52,53^, where the generated clinical text is restricted by the context (e.g., the prompts). Specifically, given a sequence of $m$ tokens ${{X}_{{pre}}=x}_{1}{x}_{2}...{x}_{m}$ as input context, the task is to generate the next $n$ continuation tokens ${{X}_{{cont}}=x}_{m+1}{x}_{m+2}...{x}_{m+n}$ until reaching the max length of 512 tokens. We generate text through iteratively sampling from the pre-trained language model GatorTronGPT one token at a time by conditioning on the preceding context:

1 $$P({x}_{{cont}}{\rm{|}}{x}_{{pre}})=\mathop{\prod }\limits_{i=m+1}^{m+n}P({x}_{i}{\rm{|}}{x}_{1}...{x}_{i-1})$$

where $P({x}_{i}|{x}_{1}\ldots {x}_{i-1})$ is the next token distribution. We adopt *Top-p* (nucleus) sampling^54^ during sampling to select words whose cumulative probability exceeds a predefined threshold *p*.

2 $$\sum _{x\in {V}^{(p)}}P(x{\rm{|}}{x}_{1:i-1})\ge p$$

where ${V}^{(p)}$ is the top-p vocabulary used to sample the next word. This approach dynamically adapts the number of words considered at each step based on their probabilities, balancing diversity and coherence of the generated text.

We set the parameter of *top-p* sampling at 0.9 and the parameter for temperature sampling at 1.2 according to our empirical assessment. We sampled the beginning 15 tokens from all sections of the de-identified notes from the MIMIC III database^22^ and generated approximately 8 million prompts. We also tried several random seeds in GatorTronGPT to generate multiple documents from one prompt. We controlled GatorTronGPT to generate a maximum length of 512 tokens.

#### Synthetic NLP model development

We applied GatorTronGPT to generate different sizes of synthetic clinical text including 1 billion, 5 billion, 10 billion, and 20 billion words of clinical text and developed corresponding synthetic NLP models, denoted as GatorTronS. Following our previous study^15^, we trained GatorTronS using the same architecture of GatorTron – a BERT architecture with 345 million parameters.

#### Comparison with existing transformer models

We compared GatorTronS models with ClinicalBERT^55^—an existing clinical transformer model and GatorTron^15^, the current largest clinical transformer model trained using >90 billion words of text, using 5 clinical NLP tasks including clinical concept extraction, medical relation extraction, semantic textual similarity, natural language inference, and question answering.

#### Turing test of text generation for healthcare settings

We randomly sampled 30 narrative sections from real-world UF Health clinical notes, including “past medical history”, “history of present illness”, “assessment/plan”, and “chief complaint”. For each of the 30 sections, we extracted the beginning 15 tokens as a seed for GatorTronGPT to generate a synthetic paragraph up to 512 tokens. We cut off the 30 real-world clinical sections to 512 tokens, removed all format information, and randomly mixed them with 30 synthetic sections written by GatorTronGPT. Two UF Health physicians (NSO, MMA) manually reviewed the 60 paragraphs of notes to evaluate: (1) linguistic readability on a 1(worst) to 9 (best) scale, (2) clinical relevance and consistency on a 1 to 9 scale, (3) determine if it was written by a human physician or GatorTronGPT. Percent agreement and Gwet’s AC_1_ were calculated to evaluate interrater reliability^56^.

### Supplementary information

Supplementary Information

## Data Availability

The benchmark datasets that support the findings of this study are available from the official websites of natural language processing challenges with Data Use Agreements. More specifically: 1. i2b2 2010, 2012 datasets and n2c2 2018, 2019 datasets: https://portal.dbmi.hms.harvard.edu/projects/n2c2-nlp/. 2. MedNLI dataset: https://physionet.org/content/mednli/1.0.0/. 3. emrQA dataset: https://github.com/panushri25/emrQA#download-dataset. 4. The Pile dataset: https://pile.eleuther.ai/. 5. UF Health IDR clinical notes are not open to the public due to patient privacy information. The GatorTronS, and GatorTron models are available as open-source resources. The synthetic clinical transformer model, GatorTronS, is available from: https://huggingface.co/UFNLP/gatortronS. The GatorTron model trained using real-world clinical text is available: https://huggingface.co/UFNLP/gatortron-base.

## Code availability

The computer codes to train GatorTronGPT models are available from: https://github.com/NVIDIA/Megatron-LM/blob/main/pretrain_gpt.py. The scripts used for data preprocessing, vocabulary training and other utilities are available from: https://github.com/uf-hobi-informatics-lab/GatorTronGPT. The computer codes to train GatorTronS models are available from: https://github.com/NVIDIA/Megatron-LM and https://github.com/NVIDIA/NeMo. The computer codes for preprocessing of text data are available from: https://github.com/uf-hobi-informatics-lab/NLPreprocessing.
