# Supplementary material for: A study of generative large language model for medical research and healthcare
Source: NPJ Digit Med. 2023 Nov 16;6:210. doi: 10.1038/s41746-023-00958-w (PMC10654385; doi:10.1038/s41746-023-00958-w)
Supplement: Supplementary file 1 — Supplementary Information [file 41746_2023_958_MOESM1_ESM.pdf]

Supplementary Information

Supplementary Table 1. Example of synthetic clinical notes written by GatorTronGPT. Examples 1, 2, and 3 passed Turing test. Examples 4, 5, 6 failed Turing test.

**Example 1.** [**NAME**] is a [**AGE**] y.o. male with a history of HTN, HLD, cardiomegaly, old MI, coronary atherosclerosis with angina, Stage III CKD, BPH, and iron-deficiency anemia who presented to [**LOCATION**] ED [**DATE**] for chest tightness, fatigue, nausea, back pain, and bilateral knee pain. The patient reports a chronic cough and nausea over the past week. Last night after dinner (without relief from tums or peppermint) he developed stomach pain and went to bed. During the early morning of [**DATE**] he awoke feeling exhausted and nauseous. His wife took his vital signs and found that his SBP was 100, which is approximately 25 points lower than normal. He noted that his breathing was [**DATE**] despite no apparent exertion during sleep. In addition, he also stated that he had less nausea while sleeping in bed compared to two nights earlier when these sxs recurred with exertion after yard work. Patient decided to come into the emergency room, thinking that his low blood pressure may be a result of dehydration. In the ED, initial vs were: T: 97.7, P: 53, BP: 122/39, R: 24, SpO2: 99% RA. Initially the patient had a low grade temp of 100.9 that responded to tylenol. EKG sinus bradycardia without evidence of ischemia. CXR did not show evidence of acute process. However, UA has evidence of UTI and initial labs show evidence of urosepsis. Patient was given one dose of ceftriaxone IV and transferred to ICU for further monitoring. At the time of transfer, vitals were: BP 128/48, HR 60's, 100% on 4L NC. Patient remained asymptomatic during this time. On ICU evaluation, patient denied any complaints and had no fever since transfer to the ICU. Pt was hypotensive in the ICU to the 70's (with concomitant bradycardia). Pt was given 500 cc bolus x2 with appropriate improvement in bp; blood pressure remained >100 SBP after bolus. Initial lactate 2.4, with subsequent lactate 1.4. Pt ruled in for NSTEMI due to cpk peak is 927 (troponin 3.44); pt placed on ASA, plavix and started on Heparin drip. Of note, patient's baseline SB
**Example 2.** Interval History: Ms [**NAME**] is a [**AGE**] yo lady whom I first met in consultation in [**DATE**]. To review briefly her past history includes a right frontal spontaneous hemorrhage in [**DATE**] secondary to untreated mild von Willebrands disease, a hospital course which was complicated by dehiscence of the bone in her frontal skull, and placement of two ventriculoperitoneal shunts. She had presented with recurrent symptomatic bradycardia to cardiology, she then developed waxing and [**NAME**] mental status which was worrisome for seizures, or a complication of her previous bleeding. EEGs were never conclusive. On exploration, she was found to have a purulent left frontal lesion; this grew Klebsiella and Prevotella (MRSA was only present on one culture). The patient underwent removal of her VPS, debridement of the left frontal lobe and endoscopic placement of both re-purpose VPS. Intraoperatively it was felt there was no acute evidence of any underlying cerebritis to the patient's presentation, rather chronic left frontal complications of her former right frontal lobectomy. Based on records the patient is highly impulsive and as a result failed a phase 1 trial of her regular ketogenic diet. Therefore she was transferred back to TICU for continued management, and repeat neuro testing. TICU course c/b tachycardia (aflutter) currently on amiodarone and diltiazem, and massive epistaxis with followup angio negative. Over past 24 hours: No acute events overnight. Hemodynamically stable, max I&O negative 500. Na trended up from 134 to 137 s/p D5.45NS (over 48hr), contiues on D10 at 20/hr; Na q4h. Subtotal transition to guanfacine. Current medications: Acetaminophen 4. Amiodarone 5. Aspirin 6. Aztreonam 7. Bacitracin Ointment 8. Bisacodyl 9. Chlorhexidine Gluconate 0.12% Oral Rinse 10. Ciprofloxacin 11. Dextrose 50% 12. Docusate Sodium (Liquid) 13. Ferrous Sulfate 14. Furosemide 15. Guanfacine 16. Heparin 17. HydrALAzine 18. Insulin 19. LeVETiracetam 20. Metoclopramide 21. Metoprolol Tartrate 22. Montel
**Example 3.** History of Present Illness: [**AGE**] y/o female with PMH CAD, ESRD on HD, HTN, DM, hx of NSCLC, breast cancer s/p resection, and idiopathic diffuse alveolar hemorrhage, presenting with oxygen saturations in 80s on routine evaluation at pulmonary and hypertension clinic on [**DATE**] with progressive oxygen desaturations while laying flat. Pt with cough and some wheeze for about 2-3 weeks after episode of diarrhea but symptoms resolved except cough until that day. She had one episode of small volume emesis prior to appointment. No GI symptoms since, no URI symptoms, no increased SOB. No hemoptysis. SOB has progressively worsened. Of note, recent R lower leg hematoma that seems to be improving, although she does have associated lightheadedness and has reportedly difficulty with walking, had decreased exercise tolerance due

to this. Recent noncontrast CT abdomen on [**DATE**] with stable interval findings. Currently, Neb therapy is unhelpful, pt does not require NRB oxygen. Transient improvement on NRB 15 l/min. L groin AV graft for HD. . Of note, pt with hx of subendocardial MI 10 yrs ago that was managed medically, hyperlipidemia, and hypertension. Pt with syncope in setting of beta blocker without further episodes. Of note, she has R pleural effusion and R sided PNA following therapeutic thoracentesis at oncology clinic in [**DATE**]. Her recent angiography for Hemodialysis showed extensive coronary vascularization through collaterals. Patient refused coronary stenting last week after she presented to [**NAME**] for likely elective [**NAME**], [**NAME **] needed to have intubation for hypoxic respiratory failure. Patient was to have had CABG last week when, over the period of one hour, she developed pain in her jaw and radiation to her jaw and went into florid pulmonary edema and hypertensive urgency which were managed using nitroglycerin/Lasix/bivalirudin. A bronchoscopy after admission showed massive bloody airways with clots but no active source of bleeding . Currently, diffuse emphysema but significant consolidation of the entire LUL appreciated on CT scan. No pulmonary artery emboli . During admission, patient's SBP ranged from the 120's to the 150's after admission. BPs have been much elevated to 180/74
**Example 4.** PLAN: 1. Lifestyle modifications: Continue daily activities. No restrictions at this time DISPO: D/C home D/C instruction form prepared and reviewed by [**NAME**] [**NAME **] [**NAME **] and given to patient. Questions addressed and answered by bedside RN. Case reviewed and discussed with attending. REHAB [**NAME **] [**NAME **] from OP [**NAME **] office ([**TELEPHONE**]. Please follow-up with pt's movement ([**NAME**] [**NAME **] [**NAME **], RN (rehab d/c coordinator) at [**TELEPHONE**]. Procedures: Phone Conversation with Occupational therapy from OPEN ZONE for independent OT eval done on [**DATE**]. Also her home visit is set for Friday. [**DATE**] Already evaluated and signed up for PPEC Per discharge from hospital at [**DATE**] Patient was in middle of her PROM stretching. She presents infant with arm out stretched in MOM lap. With several kicks/gaits movements, shows freedom of neck. Using hand in incomplete reaching at shoulder height with arm facing out. Internal rotation noted at -15 degrees and external rotation at 0 degrees. Demonstrates grip on chuck. With distraction at torso her extension can extend beyond overall back closure position , no arms crossed. Vigorous feeding with jaw movement noted. PO feeding rice cnoodle shaped cow in semi reclined seat. IMPRESSION: Early fracture type injuries and [**NAME**] on present exam. Medications on Admission: Poly-Vi-[** NAME**] 1 ml daily Tri-Vi-Sol 1 ml daily Garlic Preemie nipple off breastmilk due to gassiness Discharge Medications: None Discharge Disposition: Home with Service Facility: OPEN ZONE Discharge Diagnosis: 1. LEFT shoulder luxatio-fossa immolans Discharge Condition: MOTHER AGREES TO PRESENT INFANT FOR FURTHER EVALUTION, RESEARCH,
**Example 5.** Subjective: We had the pleasure of seeing [**NAME**] who is a [**AGE**] y.o. male being seen today for Fall Today, patient presents after follow up fall and left arm abrasion. Pt notes pain has reduced markedly but ocassional tendon rubbing/stiching when working making it worse. Sensation and motor movements and functions returned. Pt notes 2 stitches left and next Thursday se made appointment with trauma centre to get removed. Review of Systems: Review of Systems Constitutional: Negative for activity change, appetite change, fatigue, fever and unexpected weight change. Genitourinary: Negative for dysuria and frequency. Skin: Positive for wound. Psychiatric/Behavioral: Negative for agitation, behavioral problems, confusion, decreased concentration, dysphoric mood, hallucinations and sleep disturbance. History, Medications, Allergies: Past Medical History: Diagnosis Date • High cholesterol • Hypertension • Mixed hyperlipidemia [**DATE**] • OSA (obstructive sleep apnea) Was told mild. Past Surgical History: Procedure Laterality Date • FOOT SURGERY [**DATE**] • SEPTOPLASTY [**DATE**] • TESTICLE TORSION REDUCTION [**DATE**] Family History Problem Relation Age of Onset • Diabetes Mother • Stroke Mother • High Blood Pressure Mother • Cancer Mother Social History Socioeconomic History • Marital status: Married Spouse name: Not on file • Number of children: Not on file • Years of education: Not on file • Highest education level: Not on file Occupational History • Not on file Social Needs • Financial resource strain: Not on file • Food insecurity Worry: Not on file Inability: Not on file • Transportation needs Medical: Not on file Non-medical: Not on file Tobacco Use • Smoking status: Never Smoker • Smokeless tobacco: Never Used Substance and Sexual Activity • Alcohol use: Yes Alcohol/week: 20.0 standard drinks Types: 20 Cans of beer per week Frequency: 4 or more times a week Drinks per session
**Example 6.** HPI [**NAME**] is a [**AGE**] y.o. female here for Breast Problem (Fibrocystic) Pt is a new pt to breast clinic - follow up for initial evaluation. Has a history of breast problems since [**DATE**], records in chart. Long list of work-ups. H/o breast lumps that are tender as well as reportable breast masses on her bilateral breast ultrasound - Fibrocystic tissue. Currently on Micronor birth control, [**NAME **] [**NAME **] hormone replacment. Bilateral breast discomfort/swelling. Pt feels mass "sometimes" other times it is

negligible. Pt felt that removal of one of her palpable masses is more due to concern and also because she would be happy to free herself from medications that makes her gain weight. Pt's appeite is also diminished due to gastroparesis. Desires surgery for breast lumps and requests OBGYN opinion before surgery and for pt to undergo US as follows: [**DATE**] Cyst #1) simple cyst #2) probable solid lesion. We have referred her to the [**LOCATION**] clinic. ***Gynecology Consult and MRI pending *** Diabetes Mellitus (DM), Type I Assessment: -Recent diagnosis -BS Action: -Insulin adjusted, Gtt discontinued and pt. started on Glargine, Regular ACHS. Response: BS Plan: -Cont. to monitor BS -Start SSC/Cardiac heart healthy diet. Anxiety Assessment: Pt emotional last pm at [**LOCATION **] Action: -Hydrocodone dc -Trazodone -Vistaril @ HS -Revound @ HS -Ativan 0.25 @ HS Response: Pt cont to express her feelings/sorrow Plan: Cont to support patient ------ Protected Section ------ Pt in soft wrist restraint per current Clinical Leader - RN s last shift r/t high-risk of harm / pulling/falling out of bed etc. Pt HOH therefore difficult to comprehend as deaf mute. Pt difficult to ascertain under

Supplementary Table 2. Percent agreement and interrater reliability for readability.

		**Physician 1**		
		High	Low	Total
**Physician 2**	High	42	3	45
	Low	10	5	15
	Total	52	8	60

Percent agreement = 0·78, interrater reliability (Gwet's AC_1)[1] = 0·69

Supplementary Table 3. Percent agreement and interrater reliability for clinical relevance.

		**Physician 1**		
		High	Low	Total
**Physician 2**	High	44	6	50
	Low	7	3	10
	Total	51	9	60

Percent agreement = 0·78, interrater reliability (Gwet's AC_1)[1] = 0·70

Supplementary Table 4. Comparison of unigrams, bigrams, and entropy between real-world notes and synthetic notes generated by GatorTronGPT.

**Metrics**	**Real notes**	**Synthetic notes**	**Examples of new N-gram generated by GatorTronGPT**
Unigrams	4.82 million	40.43 million	radline, atyps, gadolin, bnzodzpn, barbitrt, wardname, anisocyt, poiklo, dssd, pbec
Bigrams	62.51 million	416.35 million	name titles, face arial, trigger work, thickness cavity, technique mdct, boots sq, daily rsbi, sinus ascending, resuscitate disposition, synchronously dysynchrony

Entropy	4.95	4.97	-

Supplementary References

1 Wongpakaran N, Wongpakaran T, Wedding D, Gwet KL. A comparison of Cohen's Kappa and Gwet's AC1 when calculating inter-rater reliability coefficients: a study conducted with personality disorder samples. *BMC Med Res Methodol* 2013;**13**:61. https://doi.org/10.1186/1471-2288-13-61.
